# Quality of life during the epidemic of COVID-19 and its associated factors among enterprise workers in East China

**DOI:** 10.1186/s12889-021-11414-3

**Published:** 2021-07-10

**Authors:** Xiaoxiao Chen, Qian Xu, Haijiang Lin, Jianfu Zhu, Yue Chen, Qi Zhao, Chaowei Fu, Na Wang

**Affiliations:** 1 Taizhou City Center for Disease Control and Prevention, Taizhou, 318000 Zhejiang Province China; 2 School of Public Health; Key Laboratory of Public Health Safety; NHC Key Laboratory of Health Technology Assessment, Fudan University, 130 Dong’an Road, Shanghai, 200032 China; 3 Deqing County Center for Disease Control and Prevention, Huzhou, 313299 Zhejiang Province China; 4 Faculty of Medicine, School of Epidemiology and Public Health, University of Ottawa, Ottawa, Ontario K1G 5Z3 Canada

**Keywords:** Public health, Epidemiology

## Abstract

**Background:**

The COVID-19 related lockdown and home confinement might have an important impact on the quality of life in enterprise workers. We investigated the quality of life during the epidemic in enterprise workers who just returned to work, and assessed its potential influencing factors to have a better understanding of the impact of COVID-19 epidemic lockdown and home confinement.

**Methods:**

This was a cross-sectional study of enterprise workers conducted in Deqing and Taizhou, Zhejiang Province, China. The Chinese version of EQ5D was used to assess life quality, and information about general characteristics and COVID-19 related factors was collected by a structured questionnaire, which was distributed through the social application “WeChat”. Multiple liner regression was used to investigate potential influencing factors.

**Results:**

A total of 2420 participants were enrolled, 59.5% of which worked in Deqing. About 50% of the participants reported worries about the COVID-2019 epidemic and 40.2% had a centralized or home quarantine during the epidemic. The mean EQ-5D score and VAS were 0.990 and 93.5. Multiple liner regression showed that the quality of life measures was related to physical activities (*β* = 0.006) and keeping home ventilation (*β* = 0.063) in Deqing, and were related to wearing a mask when going out (*β* = 0.014), keeping home ventilation (*β* = 0.061), other marital status (*β* = − 0.011), worry about the epidemic (*β* = − 0.005) and having a centralized or home quarantine (*β* = − 0.005) in Taizhou.

**Conclusions:**

The quality of life for returning enterprise workers in areas with different risks of COVID-19 was affected by different factors. Associated factors identified from this study would help develop proper intervention measures for enterprise workers to reduce the impact of large-scale public health events like the COVID-19 on their quality of life.

## Background

The outbreak of novel coronavirus disease 2019 (COVID-19) caused by severe acute respiratory syndrome coronavirus 2 (SARS-CoV-2) occurred in Wuhan, Hubei province, China [1, 2]. Since then, the infection has been spreading rapidly and has affected millions of people worldwide [3–5], especially the elderly and those with comorbidities [1, 6]. To prevent and control the spread of COVID-19, the Chinese central government took a series of unprecedented measures in late January 2020, including a lockdown for Wuhan, the epicenter of the epidemic, and implementing quarantine measures countrywide [7].

During the nationwide lockdown, people were socially isolated [8], and experienced negative emotions such as fear and stress [9–11] and lifestyle changes including reduced physical activity and increased sedentary time [12–14]. The lockdown due to the epidemic of COVID-19 had a negative effect on mental health for both patients with COVID-19 [15–17] and general populations [17–21], also reflected by the health-related quality of life (HRQoL) [22, 23].

To evaluate the quality of life during the long-term social isolation and lockdown caused by COVID-19 epidemic, recent researches have assessed and evaluated the quality of life using psychometric instruments (e.g. SF-18) [14] and utility instruments (e.g. EQ-5D-3L) [24] in Chinese adult populations. However, The impact of COVID-19 lockdown on the quality of life has not been well studied for working people, especially for those who stop working during the lockdown from epidemic. A recent study prompted more attention to the health of people who were not infected by virus but stopped working during the outbreak [25].

Moreover, mask use was proved to be an effective preventive strategy for health care workers to reduce the risk of infection [26, 27], which might reduce the fear and stress from COVID-19, and strategies such as keeping social contacts and healthy lifestyle, fostering self-efficacy, and information on where to get medical treatment seemed be helpful for general population [28]. However, the influencing factors or protective measures for quality of life of people who temporarily stopped working under the burden of the COVID-19 were unclear. Hence, we hypothesized that the quality of life for working people who brought the main household income, might be influenced by associated factors during the 1–2-month lockdown period and evaluated the quality of like for enterprise workers and explored its influencing factors for a better understanding of the impact of lockdown due to the COVID-19 epidemic.

To understand the impact of measures taken to prevent epidemic or infection of COVID-19 on health effects as well as the related influence factors is helpful for better-informed decisions. As the risk of new round outbreak increases, we may benefit from past experience during the epidemic, especially before the large-scale use of vaccines.

## Methods

### Study population and design

We carried out a cross-sectional study among the employees who had returned to work in Deqing and Taizhou, Zhejiang Province, China, from 5 March 2020 to 14 March 2020, the study design of which has been described previously [29, 30]. At the beginning of our investigation, there were 3 confirmed cases in Deqing and 146 cases in Taizhou, and Deqing was classified as a low risk area and Taizhou as a high risk area for COVID-19 epidemic [31]. Enterprises in each study site were contacted and voluntarily participated in the study until the targeted number of 900 subjects was reached in each area. The included enterprises were those that reopened in mid-February with the annual business turnover of 3 million U.S. dollars or above (converted from RMB). Finally, 123 of 738 enterprises in Deqing and 43 of 996 enterprises in Taizhou were included in this study, which covered the main kinds of enterprises in each area. The full-time employees of the participated enterprises who had returned to work since mid-February were eligible. An anonymous self-reported online inquiry including the EQ-5D was distributed through the application “WeChat”, which was widely used in China. A total of 2461 questionnaires were collected, and among them, 26 that were completed in less than 2 min or more than 60 min and 15 with missing were excluded, leaving 2420 observations for the current analysis.

### Sample size calculation

The initial minimum sample size was approximately estimated by the following formula. The prevalence of people without health conditions of “11,111” (*p*) in EQ5D was assumed to 0.2, according to results of previous studies in Chinese population [32, 33] and the potential negative effect of COVD-19. The margin of error (δ) and α level were set as 0.15p and 0.05, respectively. Considering the convenience sampling methods, the calculated sample size of 683 was amplified to 900 in each area.

$$ \mathrm{N}=\frac{Z_{1-\propto /2}^2\times p\left(1-p\right)}{(0.15p)^2} $$

### Measures of quality of life and influencing factors

The quality of life (QoL) was assessed by using the Chinese version EuroQol (EQ-5D-3L) [34], which has been previously validated [35–37]. The questionnaire of EQ-5D-3L consists of two parts. The first part comprises questions in five different health dimensions: 1) mobility, 2) self-care, 3) usual activities, 4) pain/discomfort, and 5) anxiety/depression. Each question has three levels of response, scored from 1 to 3 (no problem, some/moderate problems and extreme problems). The combination of three different levels for each of five dimensions has 243 possible conditions to describe the gravity of health status for participants [38]. The EQ-5D has been applied to a Chinese general population and reveals the utility values of each health condition, ranged from − 0.149 to 1.000, with higher scores indicating higher health status [39]. The second part contains a visual analogue scale (VAS) ranged from 0 (the worst health condition) to 100 (the best health condition) [38].

Mental health was assessed by using the 7-item Generalized Anxiety Disorder Scale(GAD-7) [40] for anxiety and the 9-item Patient Health Questionnaire(PHQ-9) [41] for depression. These two questionnaires have been validated and widely used in Chinese population [42–45]. Those two questionnaires were rated by a 4-point scale ranged from 0 (not at all) to 3 (nearly every day). Items were summed to derive a total score of GAD-7 (Ranged from 0 to 21) or PHQ-9 (Ranged from 0 to 27). The scores ranged 0 to 4, 5 to 9, 10 to 14, and 15 to 21 were often used to describe different categories of minimal, mild, moderate, and severe symptom of anxiety in GAD-7 [40], and 0 to 4, 5 to 9, 10 to 14, 15 to 19, and 20 to 27 were used to differentiate between none-minimal, mild, moderate, moderately severe, and severe depression in PHQ-9 [41].

Information about demographic characteristics, COVID-19 and lifestyle was collected by the self-structured questionnaire. Questions were asked on lifestyle factors, including regular alcohol drinking (> 3 times/week for ≥6 months) [46], regular cigarette smoking (> 3 times/week for ≥6 months) [47], regular tea drinking (> 3 times/week for ≥6 months) [48] and regular physical activity (> 10 min/day for ≥6 months) [49]. COVID-19 related information included quarantine status, awareness of COVID-19, self-protection measures and history of vaccination. Common chronic diseases (e.g. hypertension and diabetes) were self-reported and there was an open question for participants to report additional health conditions.

### Statistical analysis

The distributions of basic characteristics and quality of life based on EQ-5D were compared between Deqing and Taizhou by using the independent-samples t tests and the χ2 tests, or Wilcoxon and Kruskal-Wallis test when data distribution was skewed. The correlation between VAS and EQ-5D values was evaluated with the Pearson correlation coefficient. Multiple liner regression analysis was performed to assess the associations of influencing factors with quality of life using the forward approach to include important factors. Coefficient (*β*) and 95% confidence intervals (CIs) were calculated. All statistical analyses were performed in SPSS version 22.0, and an alpha level *p* ≤ 0.05 was considered to be statistically significant.

## Results

### Participant characteristics and COVID-19 related information

Among 2420 participants, 1232 (50.9%) were male and 1438 (59.5%) worked in Deqing (Table 1). One participant had COVID-19 infection and 3 had a close contact with COVID-19 patients. No participant reported having non-communicable disease (e.g. hypertension and diabetes). The participants were aged 35.9 years on average. Two thirds of participants (62.7%) lived in families with 4 to 6 members. The majority of participants were married (76.5%) and had an education of over 9 years (66.2%). Half of the participants (52.0%) reported an annual family income between 7500 to 30,000 USD (converted from RMB). Table 1 shows the distributions of the lifestyle factors and negative emotions by study site. There were no differences in the distributions of cigarette smoking, alcohol drinking, physical activity and depression between participants from Deqing and Taizhou, except for tea drinking (χ^2^ = 5.58, *p* = 0.018) and anxiety (χ^2^ = 6.52, *P* = 0.038).

**Table 1:** Characteristics of all participants from Deqing and Taizhou, 2020

Variables	Total	Deqing	Taizhou	***P*** value
	(***N*** = 2420)	(***n*** = 1438)	(***n*** = 982)	
Gender, n(%)				
Male	1232 (50.9)	714 (49.7)	518 (52.7)	0.135
Female	1188 (49.1)	724 (50.3)	464 (47.3)	
Age (years), n(%)				0.012
15–25	233 (9.7)	129 (9.0)	104 (10.6)	
25–35	994 (41.1)	622 (43.3)	372 (37.9)	
35–45	703 (29.0)	389 (27.1)	314 (32.0)	
45-	490 (20.2)	298 (20.7)	192 (19.6)	
Marital status, n(%)				0.123
Married	1852 (76.5)	1098 (76.4)	754 (76.8)	
Single	422 (17.4)	263 (18.3)	159 (16.2)	
Others	146 (6.0)	77 (5.3)	69 (7.0)	
Family members, n(%)				0.001
≤ 3 persons	739 (30.6)	467 (32.4)	272 (27.7)	
4 ~ 6 persons	1518 (62.7)	894 (62.2)	624 (63.5)	
≥ 7 persons	163 (6.7)	77 (5.4)	86 (8.8)	
Annual family income(approximately converted from RMB), n(%)				< 0.001
< 7500 USD	396 (16.4)	186 (12.9)	210 (21.4)	
7500–30,000 USD	1259 (52.0)	750 (52.1)	509 (51.8)	
≥ 30,000 USD	295 (12.2)	202 (14.0)	93 (9.5)	
Unclear	470 (19.4)	300 (21.0)	170 (17.3)	
Level of education, n(%)				< 0.001
≤ 9 years	814 (33.6)	354 (24.6)	460 (46.8)	
>9 years	1606 (66.4)	1084 (75.4)	522 (53.2)	
Smoking, n(%)				0.697
Yes	599 (24.8)	360 (25.0)	239 (24.3)	
No	1821 (75.2)	1078 (75.0)	743 (75.7)	
Alcohol drinking, n(%)				0.305
Yes	194 (8.0)	122 (8.5)	72 (7.3)	
No	2226 (92.0)	1316 (91.5)	910 (92.7)	
Tea drinking, n(%)				0.018
Yes	697 (28.8)	440 (30.6)	257 (26.2)	
No	1723 (71.2)	998 (69.4)	725 (73.8)	
Physical activity, n(%)				0.389
Yes	2029 (83.8)	1198 (83.3)	831 (84.6)	
No	391 (16.2)	240 (16.7)	151 (15.4)	
Anxiety				
Minimal	2120 (87.6)	1280 (89.0)	840 (85.5)	0.038
Mild	261 (10.8)	138 (9.6)	123 (12.6)	
Moderate/severe	39 (1.6)	20 (1.4)	19 (1.9)	
Depression				
None-minimal	1947 (80.5)	1171 (81.4)	776 (79.0)	0.589
Mild	401 (16.6)	228 (15.9)	173 (17.6)	
Moderate/severe	72 (2.9)	39 (2.7)	33 (3.3)	

There were about 50% reported worries about the COVID-2019 epidemic. Most participants knew about COVID-19 (85.4%) and about 40.1% of participants had a centralized or home quarantine during the epidemic. Almost everyone always wore a mask (97.2%) when going outside and kept home ventilation daily (99.4%). The majority of participants believed that the virus would be quickly under control (70.6%). Table 2 shows the results of a comparison between two study sites. Compared with participants in Taizhou, those workers in Deqing were more likely to have the knowledge about COVID-19 (χ^2^ = 13.01, *p* < 0.001) and to wear a mask when going out (χ^2^ = 21.26, *p* < 0.001), and were less likely to worry about COVID-19 (χ^2^ = 16.07, *p* < 0.001).

**Table 2:** Distribution of COVID −19 related questions for participants from Deqing and Taizhou, 2020

Variables		Total	Deqing	Taizhou	*P* values
		(*n* = 2420)	(*n* = 1438)	(*n* = 982)	
Centralized or home quarantine, n(%)	Yes	972 (40.2)	577 (40.1)	395 (40.2)	0.961
	No	1448 (59.8)	861 (59.9)	587 (59.8)	
Known about COVID-19, n(%)	Yes	2067 (85.4)	1259 (87.6)	808 (82.3)	< 0.001
	No	353 (14.6)	179 (12.4)	174 (17.7)	
Worried about COVID-19, n(%)	Yes	1298 (53.6)	723 (50.3)	575 (58.6)	< 0.001
	No	1122 (46.4)	715 (49.7)	407 (41.4)	
Believe the epidemic will be under control quickly, n(%)	Yes	1708 (70.6)	1022 (71.1)	686 (69.9)	0.52
	No	712 (29.4)	416 (28.9)	296 (30.1)	
Wear a mask when going out, n(%)	Yes	2352 (97.2)	1416 (98.5)	936 (95.3)	< 0.001
	No	68 (2.8)	22 (1.5)	46 (4.7)	
Wash hands frequently, n(%)	Yes	2407 (99.5)	1433 (99.7)	974 (99.2)	0.123
	No	13 (0.5)	5 (0.3)	8 (0.8)	
Keep home ventilation frequently, n(%)	Yes	2406 (99.4)	1433 (99.7)	973 (99.1)	0.07
	No	14 (0.6)	5 (0.3)	9 (0.9)	
Having influenza/pneumonia vaccination, n(%)	Yes	798 (33.0)	462 (32.1)	336 (34.2)	0.283
	No	1622 (67.0)	976 (67.9)	646 (65.8)	

### Quality of life quality based on EQ-5D

The majority of participants (93.8%) had a perfect score of “1.000” for EQ-5D. Few participants reported moderate anxiety/depression (4.0%) and moderate pain/discomfort (2.4%). Only 7 participants had problems on self-care. The utility of EQ-5D (Chinese version) showed a moderate correlation (0.312, *p* < 0.001) with the VAS. The mean EQ-5D score and VAS were 0.990 and 93.5, respectively. No gender or age difference was observed in total EQ-5D score and five dimensions. Nonparametric analysis suggested that participants in Deqing had an overall higher average EQ-5D score (Z = -2.023, *p* = 0.043) and VAS (Z = -3.235, *p* = 0.001) compared with Taizhou.

### Influencing factors for quality of life

The results of multiple liner regression for explanatory factors associated with compromised quality of life were presented in Table 3. In general, physical activity (*β* = 0.004), wearing a mask when going out (*β* = 0.009), keeping home ventilation (*β* = 0.063) were significantly associated with higher quality of life, while other marital status (*β* = − 0.006) and worrying about COVID-19 (*β* = − 0.004) were associated with lower quality of life.

**Table 3:** Liner regression analysis for factors associated with compromised quality of life in Deqing and Taizhou, 2020

Variables	***β*** (95% CI)	***p*** value
**All**		
Marital status		
Single vs. married	−0.002(−0.06, 0.001)	0.175
Other vs. married	−0.006(−0.012, −0.001)	0.019
Physical activity (yes vs. no)	0.004 (0.001, 0.008)	0.021
Worried about COVID-19 (yes vs. no)	−0.004(− 0.006, − 0.001)	0.006
Wear a mask when going out (yes vs. no)	0.009 (0.001, 0.016)	0.028
Keep home ventilation usually (yes vs. no)	0.063 (0.046, 0.079)	< 0.001
**Deqing**		
Physical activity (yes vs. no)	0.006 (0.001, 0.010)	0.009
Keep home ventilation usually (yes vs. no)	0.063 (0.035, 0.090)	< 0.001
**Taizhou**		
Centralized or home quarantine (yes vs. no)	−0.005(−0.009,-0.001)	0.031
Worried about COVID-19 (yes vs. no)	−0.005(− 0.009, − 0.001)	0.022
Wear a mask when going out (yes vs. no)	0.014 (0.004, 0.023)	0.005
Keep home ventilation usually (yes vs. no)	0.061 (0.039, 0.082)	< 0.001
Marital status		
Single vs. married	−0.004(−0.010, 0.001)	0.128
Other vs. married	−0.011(− 0.019, − 0.003)	0.010

In Deqing, physical activity (*β* = 0.006) and keeping home ventilation (*β* = 0.063) were significantly positively associated with high quality of life. In Taizhou, wearing a mask when going out (*β* = 0.014), keeping home ventilation (*β* = 0.061) had positive correlation with quality of life, while those who worried about the epidemic (*β* = − 0.005), experienced centralized or home quarantine (*β* = − 0.005) and being other marital status (*β* = − 0.011) had negative correlation with quality of life.

## DISSCUSION

This study investigated the quality of life among 2420 employees in Deqing County and Taizhou City during the lockdown of COVID-19 epidemic in March 2020, shortly after their return to work. Our study found that employees in Taizhou where epidemic was more severe, were more likely to worry about the epidemic and had lower quality of life compared with Deqing, a low risk area. The awareness of the COVID-19 was common and most people implemented some measures to protect themselves accordingly, which were also associated with a higher quality of life. Being married and having no isolation were associated with better quality of life, while worrying about the epidemic led to a reduced quality of life.

Our study showed an average EQ-5D utility value of 0.990(0. 041) and VAS score of 93.5(9.5) among enterprise workers in Deqing and Taizhou during the epidemic lockdown, respectively. Both the average EQ-5D utility value and VAS score were higher than those from studies conducted before the epidemic among Chinese adult population (0.951 and 88.0) [50, 51] and workers (0.959 and 81.1) [50, 51]. Compared with the recent researches during the epidemic, they were also higher than scores measured in patients with Alzheimer’s disease (utility: 0.62, VAS:52.9) [52], cancer patients (VAS:66.1) [10] and other general Chinese population (utility:0.949, VAS: 85.5) [24] as well as Moroccans with home confinement (utility: 0.86, VAS: 80.3) [53].

Moderate pain/discomfort and anxiety/depression were the common problems affecting quality of life in our study population, which was consist with results from recent studies [24, 53]. However, our study showed a low proportion of pain (2.4%) and anxiety/depression (4.0%), which was similar to results before the epidemic among Chinses population, especially in anxiety/depression(anxiety/depression:5.3%) [54], but lower than those in the findings of Ping Weiwei(pain:19.0%, anxiety/depression: 17.6%) and Asmaa Azizi(pain:30%, anxiety/depreesion:56%) [24, 53].

There are several possible reasons for the higher utility of EQ-ED and lower proportion of health problems (pain and anxiety/depression) observed in enterprise workers in Deqing and Taizhou during the COVID-19 pandemic lockdown. A study in Spain reported that people with chronic diseases had higher levels of depression and anxiety compared to those without [55]. Whereas, most participants aged 18 to 45 years old and no participants reported common non-communicable diseases (e.g. hypertension, diabetes). Besides, a relatively low risk of infection compared with Wuhan could reduce the negative psychological impact of quarantine and home confinement [56]. In addition, 69.4% participants lived with more than 4 family members. Most people received increased support and spiritual solace from their friends and family members, what would keep the participants away from the influence of social isolation and eased their anxiety or depression [18].

Our results indicated that marital status, physical activities, wearing a mask, keep home ventilation and worry about COVID-19 were related to HRQoL. Adequate physical activity and being married were related to the higher quality of life in our study, which was consist with the findings from some other Chinese studies [14, 33, 57]. However, worrying about COVID-19 showed a negative impact on the quality of life for enterprise workers, which was similar to the findings from recent studies [24]. Face mask use was an evidenced strategy to reduce the risk of infection from COVID- 19, which perhaps explained the protective effect on the quality of life in our study. Home ventilation perhaps had played a role in alleviating the boredom from long-term home confinement, and thereafter improved the quality of life during the lockdown.

There were different influencing factors for workers in Deqing and Taizhou. Regular physical activity was related to a better quality of life in Deqing, while centralized or home quarantine, worrying about the epidemic, wearing a mask, and marital status were associated with quality of life in Taizhou. Deqing was a low-risk epidemic area with only 3 confirmed cases of COVID-19 while Taizhou was a high-risk epidemic area with 146 confirmed cases of COVID-19 [31]. People in Deqing were less affected by COVID-19 and the lockdown mainly changed their lifestyles, which partly explained why only physical activity and keep home ventilation were associated with their quality of life. On the contrast, people in Taizhou were much stressful and were more likely worried about COVID-19 (58.6% in Taizhou vs. 50.3% in Deqing).

The main strength of our study was the relatively large sample size of 2420 participants and we assessed quality of life during the epidemic just after those participants returned to work. The present study also has limitations. First, as cross-sectional study, we did not have information on the quality of life before the epidemic and were unable to evaluate the overall effect of COVID-19 epidemic on the quality of life for enterprise workers. Second, as it was still in the period of COVID-19 epidemic control and prevention in early March in China, it was hard to conduct a survey in a random fashion. Non-random sampling was performed in this study, with a pre-set sample of 1800 participants (900 from Deqing, 900 from Taizhou), which might bring some selection bias and might not generalize the results to broader populations. In addition, due to the lack of details on job related information, we were not able to assess the association of quality of life with potential conditions that existed in the workplace.

## Conclusion

Returning enterprise workers in two areas with a different risk of COVID-19 experienced different life quality during the epidemic lockdown. Associated factors identified from this study might help develop proper intervention measures for enterprise workers in areas with different risks to improve their quality of life during such a large-scale public health event.

## Data Availability

The datasets used during the current study are available from the corresponding author on reasonable request.

## Abbreviations

COVID-19: Novel coronavirus disease 2019
SARS-CoV-2: Severe acute respiratory syndrome coronavirus 2
Qol: Quality of life
EQ-5D: EuroQol five-dimension
EQ-5D-3L: EuroQol five-dimension three-level
VAS: Visual analogue scale
*β*: Regression coefficient
CI: Confidence interval
